# Dental Pulp Mesenchymal Stem Cells Attenuate Limb Ischemia via Promoting Capillary Proliferation and Collateral Development in a Preclinical Model

**DOI:** 10.1155/2021/5585255

**Published:** 2021-08-31

**Authors:** Youfeng Li, Yuning Zhang, Hua Wang, Chengfeng Sun, Dongmei Liu, Hongying Liu, Jia He, Furong Chen, Weiting Wang, Xi Jiang, Chu-tse Wu, Yuefeng Yang

**Affiliations:** ^1^Beijing SH Biotechnology Co. Ltd., Beijing 100070, China; ^2^Department of Experimental Hematology, Beijing Institute of Radiation Medicine, Beijing 100850, China; ^3^Tianjin Institute of Pharmaceutical Research Drug Assessment Co., Ltd., Tianjin 300301, China

## Abstract

Critical limb ischemia (CLI), an end-stage manifestation of peripheral artery disease (PAD), still lacks effective therapeutic strategies. Recently, dental pulp-derived mesenchymal stem cells (DP-MSCs) have been attracting more and more attentions in therapeutic applications due to their high proliferation ability, powerful osteogenic differentiation potential, and effective anti-inflammatory effects. In this study, we compared the therapeutic effects of MSCs derived from different sources in a femoral artery-ligated preclinical ischemic model. We found that treatments with MSCs, including bone marrow- (BM-), adipose- (AD-), dental pulp- (DP-), and umbilical cord- (UC-) derived MSCs, improved limb functions, reduced inflammatory responses, increased angiogenesis, and promoted regeneration of muscle fiber. Among them, DP-MSCs and BM-MSCs produced much more impressive effects in restoring limb functions and promoting angiogenesis. The flow velocity restored to nearly 20% of the normal level at 3 weeks after treatments with DP-MSCs and BM-MSCs, and obvious capillary proliferation and collateral development could be observed. Although neovascularization was induced in the ischemic limb after ligation, MSCs, especially DP-MSCs, significantly enhanced the angiogenesis. *In vitro* experiments showed that serum deprivation improved the expression of angiogenic factors, growth factors, and chemokines in DP-MSCs and UC-MSCs, but not in BM-MSCs and AD-MSCs. However, DP-MSCs produced stronger therapeutic responses than UC-MSCs, which might be due to the higher expression of hepatocyte growth factor (HGF) and hypoxia-inducible factor-1 *α* (HIF-1*α*). We speculated that DP-MSCs might stimulate angiogenesis and promote tissue repair via expressing and secreting angiogenic factors, growth factors, and chemokines, especially HGF and HIF-1*α*. In conclusion, DP-MSCs might be a promising approach for treating CLI.

## 1. Introduction

Peripheral artery disease (PAD), an atherosclerotic arterial occlusive disease, is characterized by ischemia in the lower extremities [1]. It is estimated that PAD affects >200 million individuals worldwide, and the incidence is predicted to be double in 2050 with the increasing population of aging and obesity [2, 3]. Approximately 1% to 2% of patients with PAD will finally develop to critical limb ischemia (CLI), an end-stage manifestation of PAD always accompanied by chronic ischemic rest pain, impaired wound healing, and ulceration of the foot [4, 5]. Currently, the therapy of limb ischemia mainly relies on surgical revascularization. However, 50% of the CLI patients are not suitable for surgical revascularization due to the intricate vascular occlusions, severe comorbidity, sepsis, limb gangrene, and so on [6]. It is estimated that only about 25% of CLI patients can benefit from surgical treatments. Therefore, it is urgent to develop effective strategies to prevent amputation and to reduce death in patients with no treatment options.

Mesenchymal stem cell (MSC) transplantation has emerged as a promising approach in treating ischemic diseases, such as peripheral artery diseases, myocardial infarction, and stroke [7–10]. MSCs could home to the ischemic sites and regenerate damaged tissues via various mechanisms, such as reducing inflammation, stimulating proliferation and angiogenesis, and inhibiting apoptosis, as well as differentiating into endothelial cells [11–14]. Dental pulp-derived MSCs (DP-MSCs), which are similar to those derived from the bone marrow (BM-MSCs), own characteristics of high proliferation ability, powerful osteogenic differentiation potential, and effective anti-inflammatory effects. Recently, DP-MSCs have been attracting more and more attentions for their therapeutic potential in various diseases, and exciting results have been reported in preclinical models of retinal degeneration, acute cerebral ischemia, periodontitis, and so on [15–19].

In this study, we reported that BM-MSCs, adipose-derived MSCs (AD-MSCs), DP-MSCs, and umbilical cord- (UC-) derived MSCs (UC-MSCs) promoted angiogenesis and tissue repair in a rat limb ischemic model. Among them, DP-MSCs stimulated angiogenesis and promoted tissue repair much more effective than others, which might be attributed to ischemia- and hypoxia-induced expression of angiogenic factors, growth factors, and chemokines, especially hypoxia-inducible factor-1 *α* (HIF-1*α*) and hepatocyte growth factor (HGF).

## 2. Materials and Method

### 2.1. Mesenchymal Stem Cells (MSCs)

BM-MSCs, AD-MSCs, DP-MSCs, and UC-MSCs were isolated as previously reported, and all experimental procedures were approved by the ethics committee of the Beijing Institute of Radiation Medicine [18, 20–22]. Primary MSCs were seeded at a density of 8000 cells per cm^2^ and cultured for 4 days. After 5 rounds of expansion, cells were collected and the expression of CD11b, CD19, CD34, CD45, CD73, CD90, CD105, and HLA-DR were confirmed by flow cytometry. Moreover, the adipogenic differentiation and osteogenic differentiation of BM-MSCs, AD-MSCs, DP-MSCs, and UC-MSCs were confirmed.

### 2.2. Animal Studies

All animal experiment protocols were approved by the ethics committee of the Tianjin Institute of Pharmaceutical Research Drug Assessment Co., Ltd., and all procedures were performed in accordance with the relevant guidelines and regulations (no. 2019032001).

#### 2.2.1. Experimental Model of Limb Ischemia

Sixty 6-8-week-old Wistar rats (200 g-280 g), including 30 male and 30 female rats, were purchased from Vital River Laboratory Animal Technology Co., Ltd. (Beijing, China) and were divided into 6 groups without significant difference in weights and gender (*n* = 10 per group). Rats in the buffer, BM-MSC, AD-MSC, DP-MSC, and UC-MSC groups were anesthetized with 40 mg per kg pentobarbital sodium and then fixed in the supine position onto the operating table. Using an electric shaver to remove the hair from the hind limb, the femoral artery, vein, and nerve were isolated after groin incision. Then, the exposed femoral artery was ligated proximally to the deep femoral artery branch [23, 24]. Rats in the control group were processed in parallel to other groups but without ligation.

In 6 hours after ligation, 5 × 10^6^ BM-MSCs, AD-MSCs, DP-MSCs, and UC-MSCs in 300 *μ*l PBS or 300 *μ*l PBS (for the buffer group) were injected intramuscularly around the ischemic site of hind limbs.

#### 2.2.2. Functional Scoring

Semiquantitative assessments of limb function were performed using the function scoring system at 1, 2, 3, and 4 weeks after ligation of the left femoral artery (Table 1).

#### 2.2.3. Laser Doppler Perfusion Imaging

Immediately postoperatively before recovery from anesthesia, the perfusion was measured at the proximal side of ligation for 1 min at frequency of 17 kHz using a Vevo 770TM Imaging System (FUJIFILM VisualSonics Inc., Toronto, Canada). Moreover, at 1, 2, and 3 weeks after cell transplantation, rats were anesthetized with 40 mg/kg pentobarbital sodium for analysis of blood flow. The rats were placed on the platform, which has been prewarmed to 37°C. Excess hairs around the surgical area of the left leg were removed before imaging. Then, the perfusion was measured as described above. The perfusion ratios of the ischemic limb versus nonischemic limb were calculated.

#### 2.2.4. X-Ray Angiography

At 4 weeks after MSC transplantation, rats were anesthetized with 40 mg/kg pentobarbital sodium and fixed in the supine position. The left femoral artery was separated from the groin to the knee joint, and the proximal side was ligated. An artery catheter was inserted into the femoral artery from the distal side. Immediately after 0.6 to 0.8 ml iodoprolamine injection (BAYER, Leverkusen, Germany), the vascular network was evaluated by using a continuous photograph by X-ray using a high-frequency X-ray machine (Kaipu Electromechanical Co., Ltd., Zhengzhou, China).

#### 2.2.5. Histopathological Analysis

At 4 weeks after MSC transplantation, the muscular tissues in the ischemic area were collected after X-ray angiography. The tissues were fixed in 4% formaldehyde solution, processed, and stained with hematoxylin and eosin (H&E). Moreover, the proliferation in ischemic areas was detected by Ki67 staining (Abcam, Cambridge, United Kingdom) using immunohistochemistry (IHC). And the infiltration of inflammatory cells was analyzed by IHC using an anti-rat CD14 antibody and anti-rat CD68 antibody.

To determine vessel formation, the sections were also analyzed by immunofluorescence using an anti-rat CD31 antibody (BD Biosciences, San Jose, CA) and FITC-conjugated anti-IgG (Millipore Corporation, Bedford, MA). After staining with DAPI, CD31^+^ cells were observed using a fluorescence microscope (Olympus, Tokyo, Japan). Six random views were selected, and the number of vessels were counted.

### 2.3. Enzyme-Linked Immunosorbent Assay (ELISA)

Twenty-four or forty-eight hours after being cultured in FBS-free *α*-MEM, the culture media were collected and the VEGF in the supernatant was detected using the Human Vascular Endothelial Cell Growth Factor (VEGF) ELISA Kit (Cusabio, College Park, MD) according to the manufacturer's instruction.

### 2.4. Real-Time Reverse Transcription Polymerase Chain Reaction (RT-PCR)

Forty-eight hours after being cultured in *α*-MEM plus 10% FBS or FBS-free *α*-MEM, BM-MSCs, AD-MSCs, DP-MSCs, and UC-MSCs were collected. Total RNA was isolated, and cDNA was synthesized using the RevertAid First Strand cDNA Synthesis Kit (Thermo Fisher Scientific, Inc., Wilmington, DE). The mRNA expression of angiogenin, basic fibroblast growth factor (bFGF), HGF, HIF-1*α*, interleukin-8 (IL-8), monocyte chemotactic protein 1 (MCP-1), platelet-derived growth factor (PDGF), and vascular endothelial growth factor (VEGF) were quantified using SYBR® Premix Ex Taq™ (Tli RNaseH Plus, Takara Bio, Inc., Shiga, Japan) on a 7500 real-time PCR system (Applied Biosystems/Life Technologies, Foster City, CA). The relative expression levels were calculated by the 2^−ΔCT^ method, using *β*-actin as the control . The following primers were used: angiogenin sense 5′ctgggcgttttgttgttggtc3 and antisense 5′ ggtttggcatcatagtgctgg 3′, bFGF sense 5′ agaagagcgaccctcacatca 3′ and antisense 5′ cggttagcacacactcctttg 3′, HGF sense 5′ gctatcggggtaaagacctaca 3′ and antisense 5′ cgtagcgtacctctggattgc 3′, HIF-1*α* sense 5′gaacgtcgaaaagaaaagtctcg3′ and antisense 5′ccttatcaagatgcgaactcaca3′, IL-8 sense 5′ ttttgccaaggagtgctaaaga 3′ and antisense 5′ aaccctctgcacccagttttc 3′, MCP-1 sense 5′ cagccagatgcaatcaatgcc 3′ and antisense 5′ tggaatcctgaacccacttct 3′, PDGF sense 5′ gcaagaccaggacggtcattt 3′ and antisense 5′ ggcacttgacactgctcgt 3′, and VEGF sense 5′ agggcagaatcatcacgaagt 3′ and antisense 5′ agggtctcgattggatggca 3′.

### 2.5. Statistical Analysis

Data were presented as mean ± s.e.m. and statistically analyzed using GraphPad Prism software version 6 (GraphPad Software, San Diego, CA, USA). One-way ANOVA followed by the Bonferroni post hoc tests was performed to analyze multiple groups. Difference was considered significantly at two-sided *p* < 0.05.

## 3. Results

### 3.1. DP-MSCs Improve Limb Functions and Reduce Ischemia-Induced Damage

**Figure 1:**
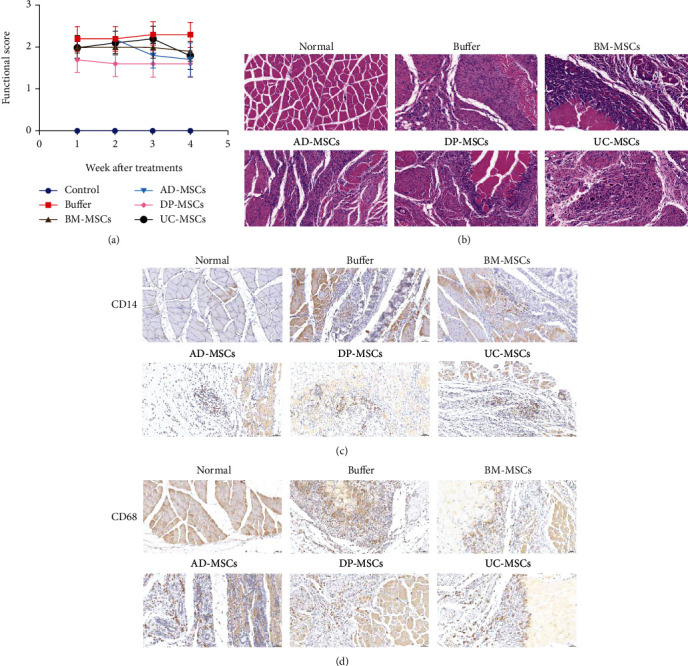
MSCs improve limb functions and reduce ischemia-induced damage. Sixty 6-8-week-old Wistar rats were divided into 6 groups: bone marrow-derived MSCs (BM-MSCs), adipose-derived MSCs (AD-MSCs), dental pulp-derived MSCs (DP-MSCs), umbilical cord-derived MSCs (UC-MSCs), buffer, and control groups. Rats in BM-MSCs, AD-MSCs, DP-MSCs, UC-MSCs, and buffer-treated groups received surgical ligation of two branches of the left femoral artery near the groin. Then, 5 × 10^6^ BM-MSCs, AD-MSCs, DP-MSCs, and UC-MSCs and 300 *μ*l PBS were injected intramuscularly into the ischemic area of hind limbs, respectively. Semiquantitative assessments of limb function were performed at 1, 2, 3, and 4 weeks after therapy, and higher scores represent much more severe damage (a). At 4 weeks after MSC transplantation, the muscular tissues in the ischemic area were collected, fixed in 4% formaldehyde solution, processed, and stained with hematoxylin and eosin (H&E). The representative images are shown in (b). The distributions of CD14^+^ cells and CD68^+^cells were analyzed by immunohistochemistry using an anti-rat CD14 antibody and anti-rat CD68 antibody. The representative images are shown in (c) and (d), respectively. Data in (a) were shown as mean ± s.e.m.

A rat model of acute limb ischemia was established by ligating the femoral artery, and MSCs derived from various tissues were injected into ischemic tissues in 6 hours after ligation. At 1, 2, 3, and 4 weeks after therapy, functional scoring showed that treatments with MSCs slightly improve the limb functions. Notably, DP-MSCs and BM-MSCs promoted recovery of limb functions much earlier than AD-MSCs and UC-MSCs (Figure 1(a)). Obvious pathological changes, such as rupture of muscle fiber, infiltration of inflammatory cells, and necrotic foci, could be detected in the buffer group at 4 weeks after ligation. MSC-treated groups significantly reduced the inflammatory responses, increased angiogenesis, and promoted regeneration of muscle fiber. Moreover, we also analyzed the infiltration of inflammatory cells by IHC in ischemic tissues. Numerous CD14^+^ cells and CD68^+^ cells could be detected in ligated areas, while MSC treatments could reduce the infiltration of CD14^+^ cells and CD68^+^ cells. Among these MSCs, DP-MSCs and BM-MSCs produced much more impressive anti-inflammatory effects (Figures 1(b)–1(d)).

### 3.2. DP-MSCs Improve Perfusion of Ischemic Limbs via Promoting Capillary Proliferation and Collateral Development

**Figure 2:**
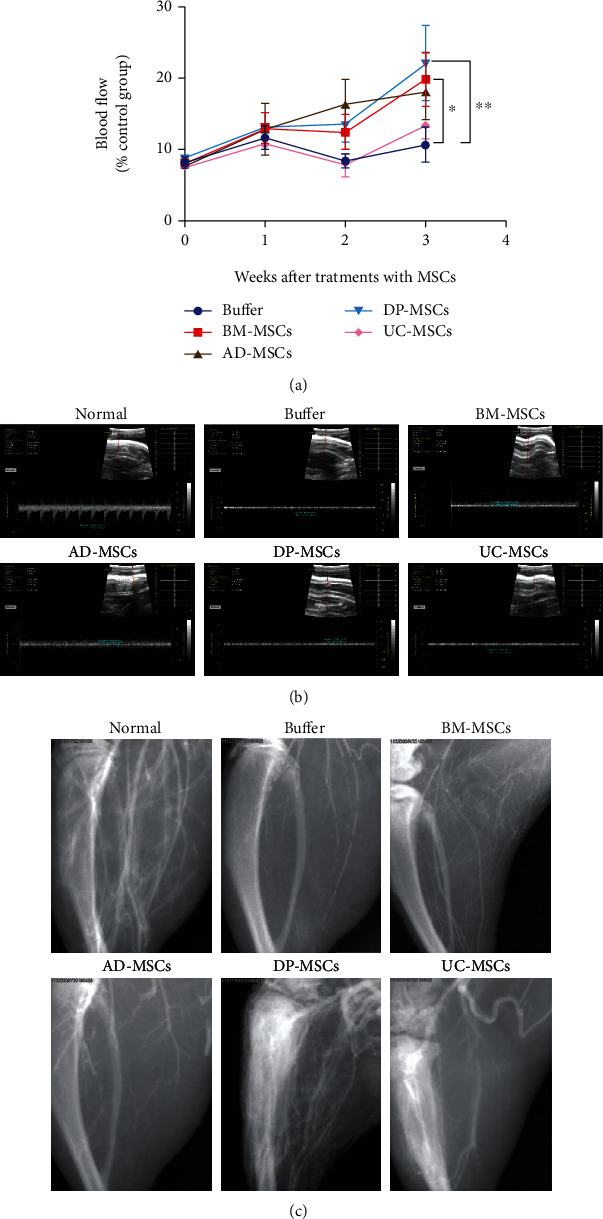
The blood perfusion and angiogenesis at ischemic limb after treatments with MSCs. Immediately after ligation and at 1, 2, and 3 weeks after cell transplantation, the blood perfusion was measured at the proximal side of ligation by laser Doppler perfusion imaging. The perfusion ratios of the ischemic limb : nonischemic limb were calculated and are presented in (a), and the representative images are shown in (b). At 4 weeks after MSC transplantation, the left femoral artery was separated from the groin to the knee joint and the proximal side was ligated. An artery catheter was inserted into the femoral artery from the distal side. Immediately after 0.6 to 0.8 ml iodoprolamine injection, the vascular network was observed through a continuous photograph by X-ray. The representative images are presented in (c). Data in (a) were shown as mean ± s.e.m. ^∗^*p* < 0.05, ^∗∗^*p* < 0.01 compared with the corresponding group.

The blood flow velocity of the femoral artery in the groin was analyzed by laser Doppler imaging in this study. The flow velocity decreased significantly at the ligated side immediately after ligation, and no obvious recovery could be observed until 3 weeks after ligation. However, MSC transplantation, especially BM-MSCs and DP-MSCs, could promote recovery of blood flow. At 3 weeks after therapy, the flow velocity in the BM-MSC and DP-MSC groups restored to nearly 20% of the normal level (*p* < 0.05 and *p* < 0.01, compared to the buffer group, respectively) (Figures 2(a) and 2(b)).

Moreover, at 4 weeks after treatment with MSCs, the microvessel density in the ischemic limbs was analyzed by angiography. Abundant blood vessels could be observed in the limbs of normal rats, while most of the blood vessels were obstructed in ligated rats. Transplantation with BM-MSCs and DP-MSCs promoted angiogenesis and significantly increased vessel density, especially microvessels (Figure 2(c)). However, no obvious angiogenesis was observed in the AD-MSC- and UC-MSC-treated groups. These results suggested that BM-MSCs and DP-MSCs might promote capillary proliferation and collateral development to restore blood supply and nutritional support in ischemic limbs.

### 3.3. DP-MSCs Promote Regeneration and Angiogenesis in Ischemic Limbs

**Figure 3:**
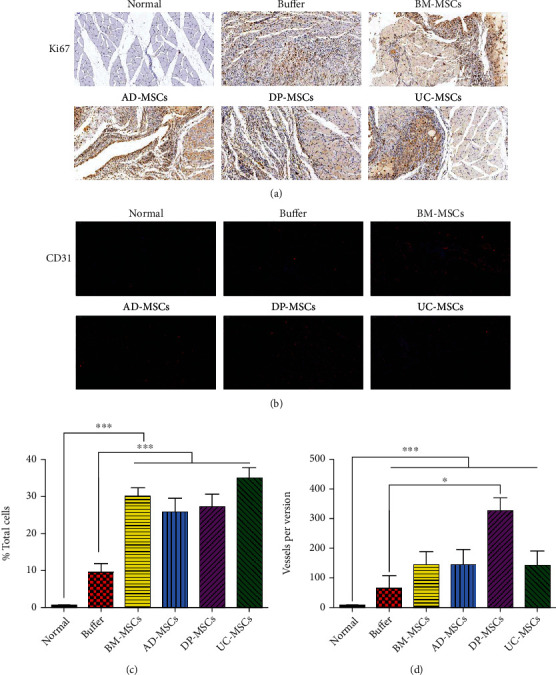
The proliferation of muscular tissues and formation of vascular lumens after treatments with MSCs. Four weeks after MSC transplantation, the muscular tissues in the ischemic area were collected, fixed in 4% formaldehyde solution, and processed. The proliferation in ischemic areas was detected by Ki67 staining using immunohistochemistry. The representative images are shown in (a). To determine vessel formation, the sections were also analyzed by immunofluorescence using an anti-rat CD31 antibody. The representative images are presented in (b). Moreover, the proliferative index and the percentage of Ki67^+^ cells were calculated (c), and the number of vascular lumens was counted (d). Data in (c) and (d) were shown as mean ± s.e.m. ^∗^*p* < 0.05, ^∗∗∗^*p* < 0.001 compared with the corresponding group.

As described above, necrosis foci could be frequently detected in ischemic limbs due to the ischemic and hypoxic microenvironment. In this study, we found that the proliferation of muscle fibers was activated in ligated limbs, which might be attributed to the self-protective or self-recovery mechanisms. MSCs could be detected in ischemic tissues at 4 weeks after engraftment (Supplementary Figure 1). Importantly, MSC treatments significantly enhanced the proliferation of muscle fibers. Although BM-MSCs and UC-MSCs produced much more impressive effects, no statistical differences could be detected among rats treated with MSCs derived from different sources (Figures 3(a) and 3(c)).

To investigate the neovascularization in ligated limbs, which plays pivotal roles in regeneration of ischemic tissues, the CD31^+^ endothelial cells were analyzed by IF. We found that vascular lumens, which were lined by CD31-positive endothelial cells, were widely distributed across the ischemic tissues in ligated limbs. Moreover, MSC transplantation enhanced neovascularization and increased the density of vascular lumens. However, a significant difference only could be detected between the DP-MSC-treated group and buffer-treated group, which suggested that DP-MSCs produced stronger effects on angiogenesis than other MSCs (Figures 3(b) and 3(d)).

### 3.4. DP-MSCs Promote Angiogenesis via Enhancing Expression of Paracrine Factors

**Figure 4:**
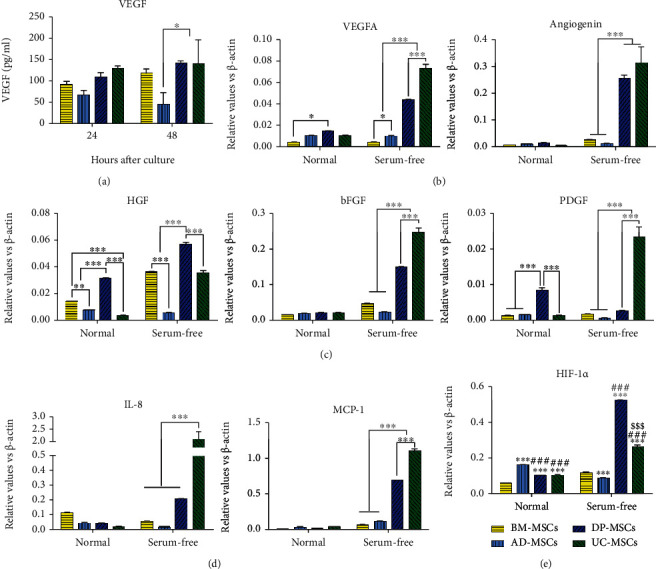
Expression of angiogenic factors in MSCs in normal or serum-free media. BM-MSCs, AD-MSCs, DP-MSCs, and UC-MSCs were seeded in a 6-well plate at a density of 8000 cells per cm^2^ and cultured with fetal bovine serum- (FBS-) free *α*-MEM at 37°C and 5% CO_2_; the culture media were collected at 24 or 48 hours after incubation. And the secretion of vascular endothelial growth factor (VEGF) was detected by ELISA (a). Forty-eight hours after being cultured in *α*-MEM plus 10% FBS or FBS-free *α*-MEM, BM-MSCs, AD-MSCs, DP-MSCs, and UC-MSCs were collected. The mRNA expression of angiogenin, basic fibroblast growth factor (bFGF), hepatocyte growth factor, hypoxia-inducible factor-1 *α* (HIF-1*α*), interleukin-8 (IL-8), monocyte chemotactic protein 1 (MCP-1), platelet-derived growth factor (PDGF), and vascular endothelial growth factor (VEGF) was analyzed by real-time reverse transcription polymerase chain reaction (RT-PCR). The relative expression levels were calculated by the 2^−ΔCT^ method, using *β*-actin as the control. Data were shown as mean ± s.e.m. ^∗^*p* < 0.05, ^∗∗∗^*p* < 0.001 compared with the corresponding groups.

Accumulating evidences suggested that MSCs produced therapeutic effects mainly through secreting a wide range of bioactive cytokines and growth factors. Here, we compared the expression of several angiogenesis closely associated growth factors in MSCs derived from different sources. Generally, serum free could induce the expression of angiogenic factors, growth factors, and chemokines in DP-MSCs and UC-MSCs. Both DP-MSCs and UC-MSCs expressed much more angiogenic regulators than AD-MSCs and BM-MSCs after serum-free treatment (Figures 4(a) and 4(b)). Interestingly, DP-MSCs expressed hepatocyte growth factor (HGF), a multiple functional cytokine which plays pivotal roles in tissue repair, at the highest level, while the expression of bFGF and PDGF in UC-MSCs were higher than that in others (Figure 4(c)). Furthermore, DP-MSCs and UC-MSCs also produced much more chemokines, such as IL-8 and MCP-1 (Figure 4(d)). In addition, DP-MSCs expressed the highest level of HIF-1*α* in a serum-free condition (Figure 4(e)). In conclusion, nutrition deprivation could induce expression of angiogenic factors, growth factors, and chemokines in DP-MSCs and UC-MSCs, but not in BM-MSCs and AD-MSCs. Importantly, DP-MSCs expressed much more HGF and HIF-1*α* than UC-MSCs, which might contribute to the stronger angiogenesis in ligated limbs.

## 4. Discussion

In past decades, MSC transplantation has emerged as a potential approach for treating CLI [8, 25, 26]. MSCs are ideal stem cells for mesodermal tissue remodeling and regeneration, due to their differentiation potential to mesoderm-derived cells, such as osteoblasts, chondrocytes, adipocytes, and vascular endothelial cells [27]. Although *in vivo* the transdifferentiation potential into endothelial cells remains controversial, it has been reported that MSCs can induce angiogenesis via various mechanisms such as paracrine [28, 29]. In this study, we found that MSC transplantations promoted tissue repair and angiogenesis effectively in a rat ischemic model. Among those MSCs, DP-MSCs could produce the strongest effects.

In recent years, MSCs have been widely used in preclinical evaluation in ischemic models. Among them, BM-MSCs are most widely used and could significantly facilitate angiogenesis [30, 31]. Studies also demonstrated that AD-MSCs promoted angiogenesis in ischemic heart and cerebral diseases and peripheral vascular disease, via secreting angiogenic factors including VEGF, bFGF, IL-6, and transforming growth factor *α* (TGF-*α*) [32, 33]. UC-MSCs have huge advantages for therapeutic applications due to their highly proliferative characteristics. It has been reported that transplantation with UC-MSCs produced significant therapeutic effects in patients with ischemic diseases [34, 35]. In this study, *vitro* data showed that serum deprivation induced much more expression of proangiogenic factor in UC-MSCs than in BM-MSCs or AD-MSCs, which was inconsistent with the therapeutic effects. These data suggested that BM-MSCs might stimulate angiogenesis of ischemic limbs via other mechanisms, or other factors restrict the therapeutic effects of UC-MSCs.

DP-MSCs were isolated from dental pulp tissue of wisdom teeth which are the last permanent teeth to develop. Therefore, DP-MSCs are more immature than BM-MSCs and possess high proliferation ability (Supplementary Figure 2). Similar to MSCs derived from other sources, DP-MSCs hold multilineage differentiation potential to osteoblasts, odontoblasts, adipocytes and chondrocytes, neurons, endothelial cells, myocytes, and hepatocytes. Until now, the therapeutic effects of DP-MSCs in bone regeneration and pulp regeneration have been demonstrated in clinical studies [36, 37]. However, it also has been reported that DP-MSCs could promote tissue repair in neurodegenerative diseases, ischemic diseases, and so on in preclinical models [16, 38]. Our *vitro* studies suggested that DP-MSC possess higher proliferation ability, more powerful osteogenic differentiation potential, and stronger anti-inflammatory and immune regulation effects than UC-MSCs (data were not shown). Moreover, our group has established well-verified platform for isolation and large-scale expansion of DP-MSCs, which could produce more than 5 × 10^10^ DP-MSCs from one donor. Researches from our group and others reported that DP-MSC could promote reconstitution of various tissues, such as bone, cementum, blood vessels, and neural tissues [18, 39–41]. In preclinical ischemic models, transplantation with DP-MSCs significantly alleviated tissue damage following cerebral or cardiac ischemia [17, 42]. However, the therapeutic effects of DP-MSCs in treating CLI are still largely unexplored. In this study, we showed that DP-MSCs could promote angiogenesis and improve perfusion much more effectively than MSCs derived from other sources.

Paracrine effects are important mechanisms for MSC-based therapy. In vitro, numerous studies demonstrated that MSCs could secret proangiogenic factors to promote migration and proliferation of endothelial cells, as well as induce capillary tube-like structure. HIF-1*α*, a key regulator in the adaptive responses to hypoxia and ischemia, is also one of the “master switches” during revascularization. HIF-1*α* could modulate the transcription of various angiogenic genes, such as transforming growth factor-*β* (TGF-*β*), angiopoietin-1, PDGF-BB, and stromal cell-derived factor-1 (SDF-1) [43]. Here, we found that serum deprivation induced the expression of HIF-1*α* obviously both in DP-MSCs and in UC-MSCs. However, DP-MSCs expressed much stronger than UC-MSCs, which might be associated with the stronger angiogenic ability of DP-MSCs in preclinical CLI. HIF-1*α* could regulate VEGF expression via activating the HIF-1*α*/VEGF pathway to elicit angiogenic regeneration [44, 45]. We found that serum deprivation upregulated the expression of VEGF in DP-MSCs at the mRNA level, but not at the protein level. HGF is a multifunctional cytokine, which plays pivotal roles in tissue repair via stimulating angiogenesis, promoting proliferation, inhibiting apoptosis, and so on. It has been reported that HGF expression increased in various injured tissues, such as the lung, kidney, and liver, and HGF modification enhanced MSC-mediated tissue repair [20, 46, 47]. In this study, we showed that HGF expression was elevated in BM-MSCs, UC-MSCs, and DP-MSCs in response to serum deprivation. However, DP-MSCs expressed much more HGF than BM-MSCs and UC-MSCs, which might contribute to the stronger neovascularization in vivo of the CLI model. Furthermore, it has been reported that MCP-1 is a potent chemotactic for macrophages and activating factor for skeletal muscle regeneration during ischemic injury. In this study, we showed that serum deprivation could significantly improve MCP-1 level both in UC-MSCs and in DP-MSCs [48].

In conclusion, BM-MSCs, AD-MSCs, DP-MSCs, and UC-MSCs effectively promoted angiogenesis and tissue repair in the preclinical CLI model. Among these cells, DP-MSCs are superior to enhance angiogenesis over others, which might be due to the strong induction of HIF-1*α* and HGF, these two pivotal growth angiogenic factors under ischemic and hypoxic microenvironment.

## Figures and Tables

**Table 1:** Functional scales.

Function score	Description
0	Normal flexion, full and fast walking
1	No toe flexion, normal but slow walking
2	No plantar flexion and no toe flexion, frequent and vigorous movement
3	Dragging, barely perceptible movement

## Data Availability

All of the data used to support the findings of this study are included within the article.

## Supplementary Materials

Supplementary 1 Supplementary Figure 1: MSC distribution in the ischemic limb. At 4 weeks after MSC transplantation, the muscular tissues in the ischemic area were collected, fixed in 4% formaldehyde solution, processed, and stained with hematoxylin and eosin (H&E). The distributions of human MSCs in the ischemic area were analyzed by immunohistochemistry using an anti-human *β*2 microglobulin antibody. The representative images were shown.

Supplementary 2 Supplementary Figure 2: proliferation ability of MSCs derived from different tissues. Primary MSCs from different sources were isolated as reported previously. After 5 rounds of expansion at a density of 8000 cells per cm2, MSCs were seeded in 96-well plates at a density of 8000 cells per cm2. The proliferation ability of MSCs was analyzed by both Cell Counting Kit-8 (CCK-8) (A) and direct cell counting (B) at 24 h, 48 h, 72 h, and 96 h.
